# On the Use of Strychnia in Intermittent Fever

**Published:** 1850-07

**Authors:** S. E. McKinley

**Affiliations:** Williamsport, Tenn.


### *On the use of Strychnia in Intermittent Fever.* By S. E. McKinley, M. D., of Williamsport, Tenn.

To the Editor of the Medical Examiner:

Dear Sir:—The object of this communication is to lay before you the experience I have had in the use of Strychnia as a remedial agent in intermittent fever. I know the ordinary treatment consists in the employment of quinine—and in ninety-nine cases in a hundred it answers, but in the hundredth it fails—given in any form or quantity, or under whatever circumstances, either during, or in the absence of either of the paroxysms, pyrexial or congestive. Calomel or blue mass may be given until ptyalism is induced, and often by such treatment alone, but more especially when followed by quinine, the attacks are postponed, but only to return in a very short time with redoubled violence. Found thus tedious in its progress, causing great emaciation and exhaustion, we ought not, without special reasons, to persist in measures calculated to lessen the powers of life, such as nauseants, bleeding, excessive sweating and repeated salivations, the ordinary treatment used in obstinate cases. And with equal propriety should we abstain from the use of that " renowned anti-periodic," when we know that it only aggravates and does not cure the disease. At the commencement it might, if properly governed, have done good and cured the patient; but now, from repeated attacks of the disease and corresponding repetition in the use of quinine, the nervous system has lost its susceptibility of being acted upon, and being brought back to a state of integrity by the use of that medicine. I have for a number of years been engaged in the active duties of my profession in many of the most malarious districts, both in the western and south-western states, and every opportunity has been afforded me of testing the various remedies for intermittent fever. But none have proved so potent in my hands as " strychnos nux vomica,"—none so cheap—none so easily administered—none that the patient will take more cheerfully—none, in fine, that acts with more certainty as a permanent cure, if persisted in for two or more weeks.

My mode of using it is to give one-sixteenth to one-eighth of a grain of strychnia every three hours, gradually augmenting the dose in proportion to the lost susceptibility. Thus we may gradually increase the quantity until, in some cases, one grain may be given three and four times per day, and without any toxical symptoms arising whatever. By pursuing the above plan, I have found my patients to recover in a very short time; and to lose the chill, in general, on the second day after commencing the medicine. I give it in pill of bread crumb. Hoping that others may meet with as satisfactory results from its use, as I have, and that it may be more used and less feared than it now is, I submit its results in my hands to the scrutiny of the profession, through the medium of the Medical Examiner.

S. E. McKinley.

P. S. Owing to the tedium of the process and the space it would require, I omit the illustration of the infinite variety of cases, with which my case-book is replete, wherein I have used the strychnia with the most marked and happy effects. The greatest number of the cases referred to were persons who contracted the seeds of their intractable malady while on a visit to, or a residence in, the "Slashes of Indiana," "Blue Ponds of Arkansas," "Black Marshes of Mississippi" and West Tennessee, and low lands on the Tennessee river. Prior to the employment of strychnia, sulphate of quinine, in the greatest number of cases, conjoined with other acknowledged tonics, such as gentian, columba, quassia, camphor, piperine, iron, &c., (after the constitutional effects of mercurials,) had been fruitlessly resorted to. Owing, probably, to the ravages the constitutional powers of the system have sustained from repeated attacks of this "Western Pest," it would seem that the nervous system has contracted a species of paralysis, amenable only to such remedies as are addressed to the system where unequivocal paralysis exists. It is well known in the western country, in malarious districts, that horses become affected—are sluggish, loathe and refuse their food—become shaggy and lose their hair—when the owners of them will at once give what they call "ox-vomit," nux vomica, and simultaneously with its action the animal revives.

